# Robotic versus freehand CT-guided radiofrequency ablation of pulmonary metastases: a comparative cohort study

**DOI:** 10.1007/s11548-023-02895-1

**Published:** 2023-04-18

**Authors:** Edward W. Johnston, Jodie Basso, Francisca Silva, Arafat Haris, Robin L. Jones, Nasir Khan, Helen Lawrence, Jakob Mathiszig-Lee, James McCall, David C. Cunningham, Nicos Fotiadis

**Affiliations:** 1 Interventional Radiology, Royal Marsden Hospital, 203 Fulham Road, London, SW36JJ UK; 2 Sarcoma Unit, Medical Oncology, Royal Marsden Hospital, 203 Fulham Road, London, SW36JJ UK; 3 Department of Anaesthesia and Perioperative Medicine, Royal Marsden Hospital, 203 Fulham Road, London, SW36JJ UK; 4 Gastrointestinal Unit, Medical Oncology, Royal Marsden Hospital, 203 Fulham Road, London, SW36JJ UK; 5 Institute of Cancer Research, 123 Old Brompton Road, London, SW73RP UK

**Keywords:** Radiofrequency ablation, Robotics, Lung neoplasms, Tomography, X-ray computed

## Abstract

**Purpose:**

Radiofrequency ablation (RFA) is a curative treatment option for small lung metastases, which conventionally involves multiple freehand manipulations until the treating electrode is satisfactorily positioned. Stereotactic and robotic guidance has been gaining popularity for liver ablation, although has not been established in lung ablation. The purpose of this study is to determine the feasibility, safety, and accuracy of robotic RFA for pulmonary metastases, and compare procedures with a conventional freehand cohort.

**Methods:**

A single center study with prospective robotic cohort, and retrospective freehand cohort. RFA was performed under general anesthesia using high frequency jet ventilation and CT guidance. Main outcomes were (i) feasibility/technical success (ii) safety using Common Terminology Criteria for Adverse Events (iii) targeting accuracy (iv) number of needle manipulations for satisfactory ablation. Robotic and freehand cohorts were compared using Mann–Whitney *U* tests for continuous variables, and Fisher’s exact for categorical variables.

**Results:**

Thirty-nine patients (mean age 65 ± 13 years, 20 men) underwent ablation of 44 pulmonary metastases at single specialist cancer center between July 2019 and August 2022. 20 consecutive participants underwent robotic ablation, and 20 consecutive patients underwent freehand ablation. All 20/20 (100%) robotic procedures were technically successful, and none were converted to freehand procedures. There were 6/20 (30%) adverse events in the robotic cohort, and 15/20 (75%) in the freehand cohort (*P* = 0.01). Robotic placement was highly accurate with 6 mm tip-to-target distance (range 0–14 mm) despite out-of-plane approaches, with fewer manipulations than freehand placement (median 0 vs. 4.5 manipulations, *P* < 0.001 and 7/22, 32% vs. 22/22, 100%, *P* < 0.001).

**Conclusions:**

Robotic radiofrequency ablation of pulmonary metastases with general anesthesia and high frequency jet ventilation is feasible and safe. Targeting accuracy is high, and fewer needle/electrode manipulations are required to achieve a satisfactory position for ablation than freehand placement, with early indications of reduced complications.

## Introduction

Radiofrequency ablation (RFA) is a minimally invasive thermal treatment strategy that can cure small lung metastases, and causes minimal damage to lung parenchyma [1] such that lung function does not change significantly following treatment [2, 3], with low postprocedural pain [4], short (usually overnight) hospital stay [5] and effective local control, around 90%, which persists up to 3 years at least [6, 7].

RFA conventionally requires multiple freehand needle manipulations until a satisfactory electrode position is achieved, and can be technically demanding, e.g., where skeletal structures make in-plane approaches impossible. Needle manipulations damage lung parenchyma, where more manipulations have been associated with higher complication rates including pneumothorax [8, 9] in up to 67% of procedures [10]. Major complication rates of around 5–10% include pneumonia, massive hemorrhage, bronchopleural fistula, air embolism, and damage to critical structures, e.g., the brachial plexus [8, 11].

A technique which offers high needle placement accuracy and reduces the number of manipulations would therefore be welcome. Navigated (stereotactic and robotic) approaches have been widely used in liver tumor ablation [12], where randomized studies comparing freehand and navigated needle placement showed significantly fewer manipulations [13], reduced needle placement time, and improved accuracy [14] over conventional freehand placement. However, use of stereotactic and robotic techniques for lung tumor ablation have not been established in the literature.

Our group has recently started a practice in robotic interventions for biopsy and ablation procedures [15]. The purpose of this study is to determine the feasibility, safety, and targeting accuracy of robotic RFA for pulmonary metastases with high frequency jet ventilation. We hypothesized that robotic guidance was feasible, safe and could produce accurate targeting, necessitating fewer needle manipulations than with freehand guidance.

## Materials and methods

### Study procedures

This study was approved by the institutional review board, and written informed consent was obtained from all patients. Procedures were performed at a single institution, (The Royal Marsden Hospital), a specialist cancer center in (London) between July 2019 and August 2022. Robotic lung RFA procedures started in August 2021, and no freehand RFA procedures were performed thereafter due to complete institutional practice change. Consecutive patients undergoing robotic lung RFA formed a “robotic cohort” where data were collected prospectively. Cases were matched with an equal number of retrospective consecutive patients meeting the eligibility criteria who underwent freehand needle placement, prior to introduction of robotic guidance to form a “freehand cohort”.

### Study participants

Inclusion criteria were (i) patients undergoing RFA of lung metastases (ii) one or more tumors < 30 mm (iii) able to undergo general anesthesia following review in preassessment clinic. Exclusion criteria were (i) energies other than RFA (ii) use of a non-robotic stereotactic device. All patients were discussed and recruited from multidisciplinary tumor board, attended by Medical and Radiation Oncologists, surgeons, pathologists, diagnostic and Interventional Radiologists where imaging, clinical, and laboratory data were reviewed, and alternative treatments considered. No follow-up was required due to focus upon feasibility, safety, and procedural technique.

### Procedures

Our team comprised (i) a minimum of one board certified Consultant Interventional Radiologist from a group of four, who performed procedures (EJ, 4 years’ experience, and NK 14 years’ experience, JMC, 19 years’ experience and NF, 17 years’ experience of lung RFA), (ii) two Radiographers who acquired images, operated the CT scanner and RF generator (iii) a Consultant Anesthetist and Anesthetic assistant who delivered the anesthetic (iv) a Nurse who cared for the patient and provided equipment. Our initial experience with robotic procedures has been reported previously [15]. We had performed 22 robotic procedures in total (7 biopsies, 15 liver ablations) before attempting robotic lung RFA.

### Preparation

After consent, a team brief was carried out and a World Health Organization safety checklist completed. Procedures were performed in a CT scanner with 64 detector rows (Siemens Definition Edge, Erlangen, DE) under general anesthesia with patients positioned to facilitate optimal electrode path. RF ground pads were applied and high frequency jet ventilation (Monsoon Acutronic Jet ventilation system III, Hirzel, CH) was used up to 200 breaths/minute to minimize respiratory excursion, with transcutaneous CO_2_ monitoring (Sentec AG, Therwill, CH) to maintain normocapnia.

### Acquisitions

For all procedures, spiral CT images were acquired in the axial plane for procedure planning and post procedure assessment without intravenous contrast using a 3 mm slice thickness with 3 mm interval for freehand procedures, and 1 mm slice interval for robotic procedures.

### Procedures

Tumor targeting in all procedures was performed using a 15 cm co-axial needle (LeVeen CoAcess, Boston Scientific, Marlborough, MA), manipulated manually until judged as satisfactory for complete transverse coverage of the index tumor by the electrode tines. For freehand procedures, needle manipulations were monitored using intermittent low dose ‘sequential’ acquisitions each time the needle was manipulated by the operator, while still in the scanner.

### Robotic procedures

Procedures were performed using a CE marked, FDA approved robotic device (Perfint MAXIO, Perfint Pvt, Chennai, IN), licensed for CT guided procedures in the thorax, abdomen and pelvis [16]. After induction of anesthesia and prior to scanning, patients were immobilized using a vacuum mattress (Klarity Vacuum Bag, OH) to reduce external motion. The device was switched on and docked, and a sterile field prepared. After scanning, DICOM images were sent to the robot which has a planning workstation where tumors were segmented, and an appropriate path selected, including out-of-plane approaches to avoid collision with the ribs if necessary. Once confirmed, the planned needle path was reproduced in three-dimensional space by the electromechanical arm using instructions sent from the workstation. A cut was made in the skin at the needle entry point (marked by a laser on the device) and the co-axial needle inserted into the patient manually by the operator via an appropriate needle guide attached to the end effector of the robotic device. A control spiral CT was then carried out, defining the first needle placement position with respect to the target tumor. The robotic device does not manipulate the needle once positioned, meaning manual adjustments can be made at this point if necessary.

### Ablation

**Fig. 1:**
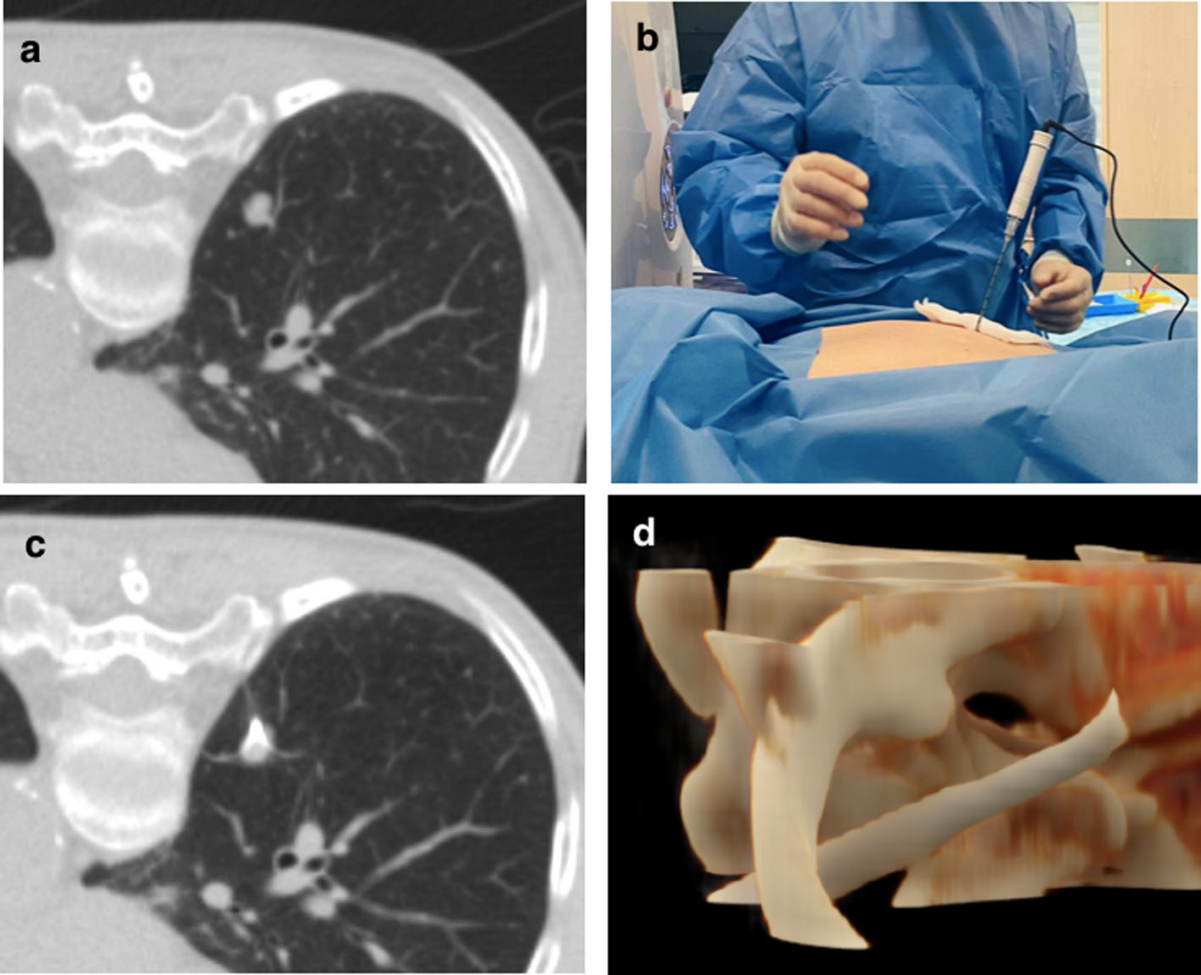
Example out-of-plane robotic radiofrequency lung ablation in a 79-year-old woman with a 12 mm solitary right lower lobe uterine leiomyosarcoma metastasis. Orbital angulation was − 1.04°, craniocaudal angulation 11.32° and target depth 45.87 mm. (**a**) Unenhanced planning axial CT image showing the tumor near the mediastinal pleural reflection, where the position of the vertebrae and ribs meant an in-plane approach required traversal of more parenchyma (**b**) photograph demonstrating the out-of-plane approach taken by the needle/electrode system (**c**) unenhanced axial CT image showing the electrode tip ablating the lesion following single co-axial needle placement (no manipulations required). (**d**) cinematic rendering demonstrating an oblique course of the co-axial needle through the posterior ribs to reach the tumor

Treatment was carried out using an ablation electrode with an array diameter chosen by the operator according to the size of the index tumor (3, 3.5 or 4 cm). An RF generator (RF2000, Boston Scientific, Marlborough, MA) delivered incremental power increase until ‘roll off’ (impedance rise) was achieved twice, according to the manufacturers’ instructions [3]. Further needle/electrode manipulation and two overlapping ablation zones were sometimes required to achieve complete tumor coverage in the longitudinal direction (of the shaft), according to operator discretion. Example images from a typical robotic ablation procedure are provided in Fig. 1. Following ablation, a repeat spiral CT was performed to assess for complications and coverage of the tumor by the ablation zone, where treatment success was defined as a penumbra of ground-glass opacification > 5 mm [17]. Delayed pneumothorax was further assessed by a chest radiograph performed 2 h post procedure and managed with a chest drain if > 2 cm or increasing in size.

### Data analysis

Data were collected on per patient, per session and per lesion levels [18]. Baseline characteristics comprised age, sex, cancer type and prior treatments per patient, concomitant liver ablation in the same session, number of target tumors per session, and lesion size, distance from pleura, and lobe.

The four main outcomes of interest were (i) technical success, defined as complete coverage of the target tumor(s) by the ablation zone(s) in both cohorts (per session), and no conversion to freehand (undocking and use of sequential acquisitions) in the robotic cohort (ii) overall safety as judged by Common Terminology Criteria for Adverse Events (CTCAE) version 5.0 [19] (per session), (iii) robotic needle placement accuracy (per lesion) iv) number of needle manipulations required to achieve satisfactory position for ablation (per lesion).

Other per session outcomes comprised: (i) RF electrode array diameter (ii) total dose length product (mGy*cm) (iii) pneumothorax, as judged on both post procedure CT and 2-h chest radiograph (iv) chest drain insertion and (v) duration of hospital admission. Other per lesion outcomes comprised: (i) number of overlapping ablation zones (ii) actual procedure time (total procedure time minus anesthetic and patient positioning time, from topogram to completion CT). (iii) length of pulmonary transgression (iv) Orbital angulation, craniocaudal angulation, and target depth for robotic procedures.

### Image analysis

**Fig. 2:**
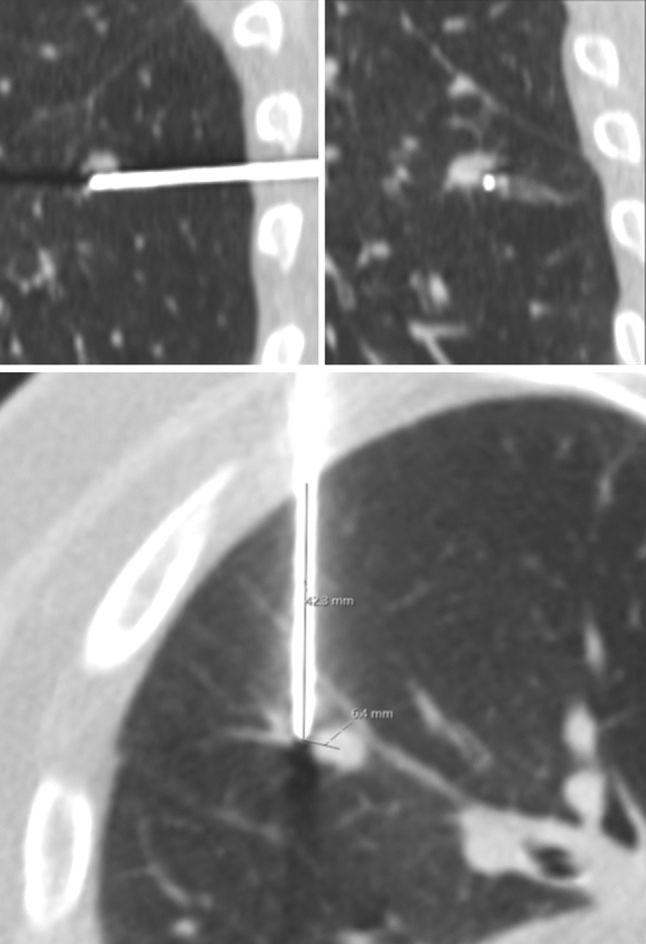
Unenhanced multiplanar reformat CT images of an 85-year-old man undergoing ablation of a left lower lobe sigmoid cancer lung metastasis. Images show measurement of first co-axial needle placement tip-to-target distance of 6.4 mm, and a parenchymal transgression length of 42 mm. N.B. This degree of error may be tolerable for satisfactory ablation due to array diameter of 3 cm or greater

**Fig. 3:**
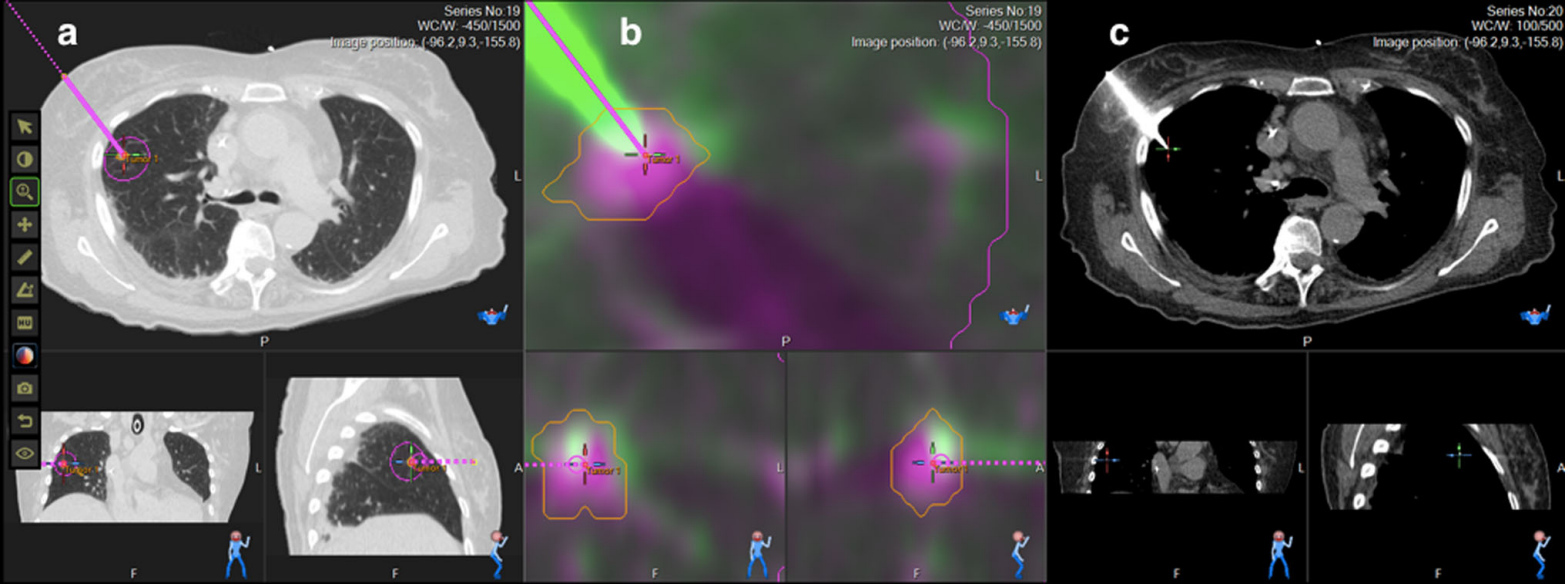
Example images from the robotic workstation, during a radiofrequency lung ablation in a 76-year-old woman with a 6 mm rectal cancer lung metastasis in the right upper lobe. (**a**) unenhanced planning CT with multiplanar reformats, showing the planned needle path in magenta (**b**) fused planning and needle placement CTs with multiplanar reformats, showing the planned needle path in magenta, and actual needle placement in solid green (**c**) multiplanar reformats of needle placement CT

DICOM images were analyzed on a PACS workstation (IDS7, SECTRA, Linköping, SWE) by a radiology fellow (AH, in training, 9 years’ experience of image analysis) blinded to all other clinical variables and unaware of the study purpose. Needle tip-to-target distance following first needle placement was measured in the robotic cohort using multiplanar reformats, and the length of pulmonary transgression was measured in both cohorts (Fig. 2). In the robotic cohort, planning and first placement CTs were fused, and standardized first needle placement targeting errors measured by an Interventional Radiologist (EJ) using the robotic workstation (Euclidian, longitudinal, lateral and angular errors between planned and actual needle paths [13, 20]). An example of fused images is provided in Fig. 3. N.B. these data were unavailable for the freehand cohort due to use of sequential acquisitions for first needle placement which did not always include the needle tip and target.

Patient age, sex, cancer type, number of prior treatments (chemotherapy lines, radiotherapy and surgery), duration of hospital admission, and complications were collected from electronic hospital records, and all other data were obtained from DICOM images and procedure reports.

### Statistical analysis

#### Study size

No formal prospective sample size calculation was carried out for this initial study focusing upon safety and feasibility. However, a post-hoc power calculation was performed for differences in the number of needle manipulations required for adequate ablation between the robotic versus freehand cohorts using G*Power 3.1 (Universität Düsseldorf, DE). Using a two tailed Mann–Whitney test at an alpha of 0.05, with 20 in each cohort and an effect size of 1.81 (calculated from the mean and standard deviation of both cohorts), our power was > 0.99.

#### Analysis

Data were analyzed using GraphPad Prism 9.4.1 (GraphPad, San Diego, Calif). All data were checked for normality using the Shapiro–Wilk test. Differences in continuous and ordinal variables were compared using independent sample *t* tests or Mann–Whitney *U* tests, and contingency tables were compared using Fisher’s exact testing. Both cohorts were compared in terms of baseline characteristics, to assess for potential confounding variables.

Statistical significance was set at *P* < 0.05. There were no missing data, apart from the single patient excluded from the study due to lack of available postprocedural images to carry out the relevant analyses.

## Results

### Patient cohorts

**Fig. 4:**
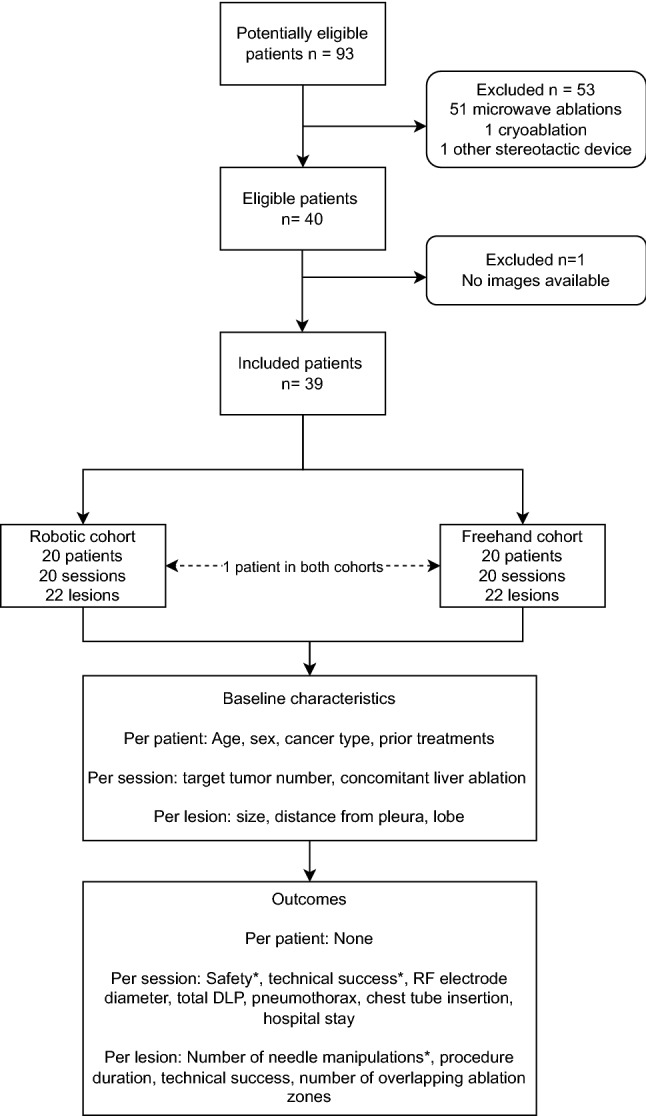
Flow diagram of study methods. The main outcomes of interest are indicated with an asterisk. Accuracy metrics were also calculated for the robotic cohort. DLP = dose length product. RF = radiofrequency

After exclusion of 53 patients who did not meet the eligibility criteria and one patient without available images, thirty-nine patients, mean age 65 ± 13 years (20 men) were treated in 40 RFA sessions for 44 lung metastases between July 2019 and August 2022, one session per patient apart from one patient who was treated in two sessions (contralateral lungs) in both the robotic and freehand cohorts, to give *n* = 20 in both in both cohorts. A summary recruitment flow diagram is shown in Fig. 4. Per patient, per session and per lesion baseline characteristics for the two cohorts are provided in Table 1, and no differences between the cohorts reached statistical significance.

**Table 1:** Baseline characteristics (per patient, per session and per lesion)

Characteristic	Robotic cohort	Freehand cohort	*P* value
*Per patient*	*n* = 20	*n* = 20	
Age, years—median (IQR)	72 (50–85)	61 (55–73)	0.42
Sex			0.75
Male—*n* (%)	10 (50)	12 (60)	
Female—*n* (%)	10 (50)	8 (40)	
Cancer type			> 0.99
CRC—*n* (%)	13 (65)	13 (65)	
Other gastrointestinal—*n* (%)	2 (10)	1 (5)	
Sarcoma—*n* (%)	3 (15)	5 (25)	
Other—*n* (%)	2 (10)	1 (5)	
Lines chemotherapy—median (IQR)	2 (1–2.8)	1.5 (1–2.8)	0.73
Radiotherapy—*n* (%)	11 (55)	7 (35)	0.34
Surgery—*n* (%)	18 (90)	17 (85)	> 0.99
*Per session*	*n* = 20	*n* = 20	
Concomitant liver ablation—*n* (%)	5 (25)	4 (20)	> 0.99
Target tumors			> 0.99
1—*n* (%)	18 (90)	18 (90)	
2—*n* (%)	2 (10)	2 (10)	
*Per lesion*	*n* = 22	*n* = 22	
Size, mm—median (IQR)	10 (6–15)	12 (10–29)	0.07
Distance from pleura, mm-median (IQR)	9.5 (0.8–16)	6.0 (2–11)	0.48
Lobe			0.66
RUL—*n* (%)	6 (27)	10 (45)	
RML—*n* (%)	3 (14)	1 (5)	
RLL—*n* (%)	6 (27)	4 (18)	
LUL—*n* (%)	2 (9)	3 (14)	
LLL—*n* (%)	5 (23)	4 (18)	

*CRC* colorectal cancer, *IQR* interquartile range, *LLL* left upper lobe, *LUL* left upper lobe, *RLL* right lower lobe, *RML* right middle lobe, *RLL* right lower lobe

### Main results

#### Technical success

All twenty (100%) of procedures were technically successful in both cohorts, and no robotic procedures were converted to freehand.

#### Safety

Six adverse events in the robotic cohort were pneumothorax of which four were managed conservatively (mild, grade I) and 2 were managed with chest drain insertion (moderate, grade II). There were fifteen session-level adverse events in total in the freehand cohort, of which eleven were managed conservatively (grade I), with ten pneumothoraces, and one instance of mild dyspnea. Three pneumothoraces were managed with drain insertion (grade II), and the single grade III adverse event was a multiterritory embolic stroke with good functional recovery following 10 days’ hospital admission and ongoing rehabilitation. Differences in adverse events reached statistical significance (*P* = 0.01).

While the percentage of pneumothoraces was considerably lower in robotic cohort (30% vs. 65%), the differences did not reach statistical significance (*P* = 0.06), nor did chest drain insertion (2 vs. 3, *P* > 0.99).

#### Robotic needle placement

Twenty-two needle insertions had a median orbital angulation of − 0.78° (range − 37.8 to 68.2°), craniocaudal angulation 0° (− 10.7 to 19.6°) and target depth 73.5 mm (range 45.2–118.7 mm). Median tip-to-target distance was 6.0 mm (0–14.0 mm). Euclidian error was 5.1 mm (0–12.5 mm), lateral error was 4.0 mm (0–8.0 mm), longitudinal error was 3.3 mm (0–10.6 mm), and angular error was 5.6° (0–10.9°).

#### Needle insertion

**Fig. 5:**
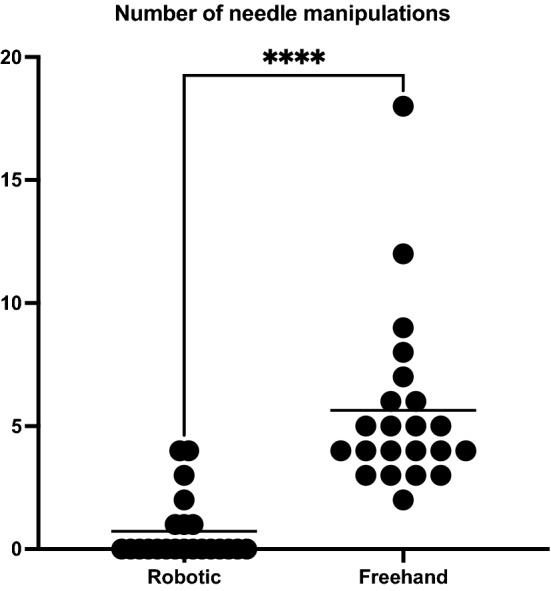
Number of needle manipulations required to gain an acceptable position of the co-axial needle prior to ablation in the robotic and freehand cohorts

There were fewer needle manipulations in the robotic versus the freehand cohort, median 0 versus 4.5, *P* < 0.001, where 7/22 (32%) required manipulation in the robotic cohort versus 22/22 (100%) in the freehand cohort (Fig. 5). Smaller array diameter electrodes were used in the robotic versus freehand cohort (3 vs. 3.5 cm, *P* < 0.001). A summary of results comparing technical outcomes between robotic versus freehand procedures is shown in Table 2.

**Table 2:** Outcomes of robotic versus freehand procedures

Outcome	Robotic cohort	Freehand cohort	*P* value
*Per session*	*n* = 20	*n* = 20	
Overall adverse events (CTCAE)—*n* (%)	6 (30)	15 (75)	0.01
None—*n* (%)	14 (70)	5 (25)	
Grade 1—*n* (%)	4 (20)	11 (55)	
Grade 2—*n* (%)	2 (10)	3 (15)	
Grade 3—*n* (%)	0	1 (5)	
Grade 4—*n* (%)	0	0	
Grade 5—*n* (%)	0	0	
Technical success—*n* (%)	20 (100)	20 (100)	> 0.99
RF electrode diameter—median (IQR)	3 (3–3.5)	3.5 (3.5–4)	0.001
Total DLP (mGy*cm)—median (IQR)	505 (254–845)	549 (303–545)	0.76
Pneumothorax	6 (30)	13 (65)	0.06
Chest drain insertion	2 (10)	3 (15)	> 0.99
Hospital stay (days)—median (IQR)	1 (1–1.75)	1 (1–1.75)	0.92
*Per lesion*	*n* = 22	*n* = 22	
Number of needle manipulations—median (IQR)	0 (0–1)	4.5 (3.8–6.3)	< 0.001
Number requiring manipulation—*n* (%)	7 (32)	22 (100)	< 0.001
Pulmonary transgression (mm)—median (IQR)	28.5 (22–48)	37 (20–46)	0.97
Number of overlapping ablation zones			0.74
1—*n* (%)	17	15	
2—*n* (%)	5	7	
Actual procedure time (mins)—median (IQR)	29.0 (22–39.5)	39.5 (28–48)	0.06

*CTCAE* Common Terminology Criteria for Adverse Events, *DLP* dose length product, IQR interquartile range, *RF* radiofrequency

## Discussion

Here we assessed the feasibility, safety, and targeting accuracy of robot guided Radiofrequency ablation (RFA) for pulmonary metastases. All our attempted robotic procedures were technically successful (20/20, 100%), with fewer complications (6 vs. 15/20, *P* = 0.01) and needle manipulations (0 vs. 4.5, *P* < 0.001) than a similar retrospective cohort of freehand procedures, despite relatively small sample size.

Procedures were successfully performed with relatively little prior experience of robotic interventions, meaning the learning curve is short. However, since the MAXIO robot assumes no movement of the target tumor, we made all efforts to keep the time between acquisition and needle insertion as short as possible to minimize target movement, including preparing the sterile field, and opening equipment before scanning. The speed of positioning the electromechanical arm is a potential advantage of robotic over manually operated stereotactic instruments, and while intermittent endotracheal tube disconnection has been successfully used in liver ablation [21], high frequency jet ventilation can minimize atelectasis and derecruitment as a source of target movement and obscuration. Importantly, jet ventilation is used continuously following induction of anesthesia and so the reduced degree of target movement remains consistent from planning to needle insertion and treatment. Although median actual procedure time was shorter in the robotic cohort (29 vs. 39.5 min), differences did not reach statistical significance (*P* = 0.06).

While the proportion of pneumothoraces was also lower in the robotic cohort (30 vs. 65%), differences did not reach statistical significance (*P* = 0.06), nor did drain insertion (10 vs. 15%, *P* > 0.99). Pneumothorax rates vary widely in the literature, between 11 and 67% [10, 22], and the ability to compare proportions using two different techniques in similar populations at a single center is a strength of this study. A handful of studies have shown that traversal of a greater length of lung parenchyma is associated with higher rates of pneumothorax [23–26], likewise fissure traversal, and ablation zones which encompass pleura [27]. Since all these factors can be influenced by careful multiplanar planning, including out-of-plane needle trajectories to navigate through skeletal structures (− 11 to + 20° in this study), robotic approaches hold significant potential in reducing lung ablation related complications.

Although robotic lung tumor ablation is poorly established in the literature, our results are similar to stereotactic liver ablation, were our Euclidian error of 5.1 mm is very similar to the pooled error of 5.3 mm in a recent meta-analysis [12]. The high level of trust we developed in our technique meant we used smaller array diameter electrodes than in freehand procedures (3.0 vs. 3.5 mm, *P* = 0.001), without a need for more overlapping ablation zones (*P* > 0.99), although broad umbrella electrode configuration means a few millimeters of imprecision can usually be tolerated. Our readjustment rate of 32% is in accordance with Beyer et al., who repositioned applicators in 41% (13/34) of liver ablations using the same device [28].

We found that radiation dose was not slightly lower for robotic versus freehand procedures (mean DLP 505 vs. 549 mGy*cm respectively, *P* = 0.76) which is unlike liver ablation, where radiation dose is usually lower with navigated procedures [12]. This could be explained by lung ablation being approximately half to a quarter of the dose of liver ablation [12], where single lesions are targeted by single applicators, using a few additional low dose sequential acquisitions for freehand needle adjustments.

The limitations to this study include retrospective data collection for the freehand cohort, meaning we were unable to make first needle placement accuracy measurements. A prospective randomized study would enable these data to be collected[13, 14], and would also control for confounding [29]. While both cohorts similar in baseline characteristics, lesion size was skewed by three lesions nearly 3 cm in the freehand cohort which likely reflects random variation with small sample size. However, this initial study focused upon feasibility, safety, and targeting accuracy and achieved high power for the number of needle manipulations versus a similar cohort of patients who underwent freehand needle/electrode placement.

Further prospective, randomized studies should be performed in more than one center, powered for clinically relevant endpoints, e.g., complications including pneumothorax. Other ablation energies, and stereotactic devices could be investigated, although we decided to first develop robotic RFA due to its sharp, rigid co-axial needle, lower cost, and high rates of local control without evidence of inferiority versus microwave ablation [4, 30].

In summary, robotic radiofrequency ablation of pulmonary metastases with general anesthesia and high frequency jet ventilation is feasible and safe. Targeting accuracy is high, and fewer needle/electrode manipulations are required than freehand placement to achieve a satisfactory position for ablation, with early indications of reduced complications.

## Abbreviations

CI: Confidence interval
CTCAE: Common Terminology Criteria for Adverse Events
IQR: Interquartile range
RFA: Radiofrequency ablation

## References

[1] Delpla A, de Baere T, Varin E, Deschamps F, Roux C, Tselikas L (2021). Role of thermal ablation in colorectal cancer lung metastases. Cancers (Basel).

[2] Lencioni R, Crocetti L, Cioni R, Suh R, Glenn D, Regge D, Helmberger T, Gillams AR, Frilling A, Ambrogi M, Bartolozzi C, Mussi A (2008) Response to radiofrequency ablation of pulmonary tumours: a prospective, intention-to-treat, multicentre clinical trial (the RAPTURE study). 10.1016/S1470-2045(08)70155-4 10.1016/S1470-2045(08)70155-4 18565793

[3] De Baère T, Palussière J, Aupérin A, Hakime A, Abdel-Rehim M, Kind M, Dromain C, Ravaud A, Tebboune N, Boige V, Malka D, Lafont C, Ducreux M (2006). Midterm local efficacy and survival after radiofrequency ablation of lung tumors with minimum follow-up of 1 year: prospective evaluation. Radiology.

[4] Macchi M, Belfiore MP, Floridi C, Serra N, Belfiore G, Carmignani L, Grasso RF, Mazza E, Pusceddu C, Brunese L, Carrafiello G (2017). Radiofrequency versus microwave ablation for treatment of the lung tumours: LUMIRA (lung microwave radiofrequency) randomized trial. Med Oncol.

[5] Chen S, Yang S, Xu S, Dong S (2020). Comparison between radiofrequency ablation and sublobar resections for the therapy of stage I non-small cell lung cancer: a meta-analysis. PeerJ.

[6] Nguyenhuy M, Xu Y, Maingard J, Barnett S, Kok HK, Brooks M, Jhamb A, Asadi H, Knight S (2022). A systematic review and meta-analysis of patient survival and disease recurrence following percutaneous ablation of pulmonary metastasis. Cardiovasc Intervent Radiol.

[7] Hasegawa T, Takaki H, Kodama H, Yamanaka T, Nakatsuka A, Sato Y, Takao M, Katayama Y, Fukai I, Kato T, Tokui T, Tempaku H, Adachi K, Matsushima Y, Inaba Y, Yamakado K (2020). Three-year survival rate after radiofrequency ablation for surgically resectable colorectal lung metastases: a prospective multicenter study. Radiology.

[8] Kashima M, Yamakado K, Takaki H, Kodama H, Yamada T, Uraki J, Nakatsuka A (2011). Complications after 1000 lung radiofrequency ablation sessions in 420 patients: a single center’s experiences. Am J Roentgenol.

[9] Anderson JM, Murchison J, Patel D (2003). CT-guided lung biopsy: factors influencing diagnostic yield and complication rate. Clin Radiol.

[10] Baère T De, Aupérin A, Deschamps F, Chevallier P, Gaubert Y, Boige V, Fonck M, Escudier B, Palussiére J (2015) Radiofrequency ablation is a valid treatment option for lung metastases: experience in 566 patients with 1037 metastases. 10.1093/annonc/mdv037 10.1093/annonc/mdv037 PMC4405279 25688058

[11] Li G, Xue M, Chen W, Yi S (2018). Efficacy and safety of radiofrequency ablation for lung cancers: a systematic review and meta-analysis. Eur J Radiol.

[12] Tinguely P, Paolucci I, Ruiter SJS, Weber S, de Jong KP, Candinas D, Freedman J, Engstrand J (2021). Stereotactic and robotic minimally invasive thermal ablation of malignant liver tumors: a systematic review and meta-analysis. Front Oncol.

[13] Heerink WJ, Ruiter SJS, Pennings JP, Lansdorp B, Vliegenthart R, Oudkerk M, de Jong KP (2019). Robotic versus freehand needle positioning in CT-guided ablation of liver tumors: a randomized controlled trial. Radiology.

[14] Beyer LP, Pregler B, Nießen C, Schicho A, Haimerl M, Jung EM, Stroszczynski C, Wiggermann P (2016). Stereotactically-navigated percutaneous irreversible electroporation (IRE) compared to conventional IRE: a prospective trial. PeerJ.

[15] Johnston EW, Basso J, Winfield J, McCall J, Khan N, Messiou C, Koh DM, Fotiadis N (2022). Starting CT-guided robotic interventional oncology at a UK centre. Br J Radiol.

[16] Koethe Y, Xu S, Velusamy G, Wood BJ, Venkatesan AM (2014). Accuracy and efficacy of percutaneous biopsy and ablation using robotic assistance under computed tomography guidance: a phantom study. Eur Radiol.

[17] Venturini M, Cariati M, Marra P, Masala S, Pereira PL, Carrafiello G (2020). CIRSE standards of practice on thermal ablation of primary and secondary lung tumours. Cardiovasc Intervent Radiol.

[18] Puijk RS, Ahmed M, Goldberg SN, Meijerink MR (2021). Consensus guidelines for the definition of time-to-event end points in image-guided tumor ablation: results of the sio and datecan initiative. Radiology.

[19] Cancer Therapy Evaluation Program (CTEP) (2017) Common Terminology Criteria for Adverse Events (CTCAE).v.5.0 [5x7]. Cancer Ther Eval Progr 155

[20] Widmann G, Stoffner R, Sieb M, Bale R (2009). Target registration and target positioning errors in computer-assisted neurosurgery: proposal for a standardized reporting of error assessment. Int J Med Robot Comput Assist Surg.

[21] Schullian P, Johnston E, Laimer G, Putzer D, Eberle G, Amann A, Effenberger M, Maglione M, Freund MC, Loizides A, Bale R (2020). Frequency and risk factors for major complications after stereotactic radiofrequency ablation of liver tumors in 1235 ablation sessions: a 15-year experience. Eur Radiol.

[22] Kennedy SA, Milovanovic L, Dao D, Farrokhyar F, Midia M (2014). Risk factors for pneumothorax complicating radiofrequency ablation for lung malignancy: a systematic review and meta-analysis. J Vasc Interv Radiol.

[23] Zhu JC, Yan TD, Glenn D, Morris DL (2009). Radiofrequency ablation of lung tumors: feasibility and safety. Ann Thorac Surg.

[24] Gillams AR, Lees WR (2007). Analysis of the factors associated with radiofrequency ablation-induced pneumothorax. Clin Radiol.

[25] Hiraki T, Tajiri N, Mimura H, Yasui K, Gobara H, Mukai T, Hase S, Fujiwara H, Iguchi T, Sano Y (2006). Pneumothorax, pleural effusion, and chest tube placement after radiofrequency ablation of lung tumors: incidence and risk factors. Radiology.

[26] Nour-Eldin N-EA, Naguib NNN, Saeed A-S, Ackermann H, Lehnert T, Korkusuz H, Vogl TJ (2009). Risk factors involved in the development of pneumothorax during radiofrequency ablation of lung neoplasms. Am J Roentgenol.

[27] Xu S, Qi J, Li B, Bie ZX, Li YM, Li XG (2021). Risk prediction of pneumothorax in lung malignancy patients treated with percutaneous microwave ablation: development of nomogram model. Int J Hyperth.

[28] Beyer LP, Pregler B, Niessen C, Dollinger M, Graf BM, Müller M, Schlitt HJ, Stroszczynski C, Wiggermann P (2016). Robot-assisted microwave thermoablation of liver tumors: a single-center experience. Int J Comput Assist Radiol Surg.

[29] Zabor EC, Kaizer AM, Hobbs BP (2020). Randomized controlled trials. Chest.

[30] Yuan Z, Wang Y, Zhang J, Zheng J, Li W (2019). A meta-analysis of clinical outcomes after radiofrequency ablation and microwave ablation for lung cancer and pulmonary metastases. J Am Coll Radiol.

